# Supplementary material for: Defining the location of promoter-associated R-loops at near-nucleotide resolution using bisDRIP-seq
Source: eLife. 2017 Oct 26;6:e28306. doi: 10.7554/eLife.28306 (PMC5705216; doi:10.7554/eLife.28306)
Supplement: Transparent reporting form [file elife-28306-transrepform.pdf]

1st Floor
24 Hills Road
Cambridge CB2 1JP, UK

P 01223 855340
W elifesciences.org
T @elife

eLife's transparent reporting form

We encourage authors to provide detailed information *within their submission* to facilitate the interpretation and replication of experiments. If you have any questions, please contact us: editorial@elifesciences.org.

Sample-size estimation

- You should state whether an appropriate sample size was computed when the study was being designed
- You should state the statistical method of sample size computation and any required assumptions
- If no explicit power analysis was used, you should describe how you decided what sample (replicate) size (number) to use

Please outline where this information can be found within the submission (e.g., page numbers or figure legends), or explain why this information doesn't apply to your submission:

Since bisDRIP-seq is a new technique, we lacked sufficient information to determine the appropriate number of samples. As such, information regarding appropriate sample size is not included in this submission.

Replicates

- You should report how often each experiment was performed
- You should include a definition of biological versus technical replication
- The data obtained should be provided and sufficient information should be provided to indicate the number of independent biological and/or technical replicates
- If you encountered any outliers, you should describe how these were handled
- Criteria for exclusion/inclusion of data should be clearly stated
- High-throughput sequence data should be uploaded before submission, with a private link for reviewers provided (these are available from both GEO and ArrayExpress)

Please outline where this information can be found within the submission (e.g., page numbers or figure legends), or explain why this information doesn't apply to your submission:

eLife Sciences Publications, Ltd is a limited liability non-profit non-stock corporation incorporated in the State of Delaware, USA, with company number 5030732, and is registered in the UK with company number FC030576 and branch number BR015634 at the address 1st Floor, 24 Hills Road, Cambridge CB2 1JP | August 2014

eLIFE
1st Floor
24 Hills Road
Cambridge CB2 1JP, UK
P 01223 855340
W elifesciences.org
T @elife

The number of control-treated bisDRIP-seq experiments performed is mentioned on pages 5 and 22, as well as the figure legends of figures 1-6. The number of triptolide-treated bisDRIP-seq experiments performed is mentioned on pages 22 and 27 and the figure legends of figures 2-5.

We clarify the nature of our replicates (biological vs technical) on page 22.

Determination of which Gencode transcription start sites to use in our full TSS list is described on pages 36-37. For promoter ranking, we excluded inactive promoters and removed promoters that had zero bisDRIP-seq score in one or more samples as described on pages 40-41. For later metaplot analysis, inactive promoters were excluded for sense R-loop analysis as described on page 12 and 37. For intron-exon metaplot analysis, inclusion of exon-intron junctions, and the associated transcription start sites, was described on pages 38-39.

A private link to high-throughput sequence data is provided on page 43.

Statistical reporting

- Statistical analysis methods should be described and justified
- Raw data should be presented in figures whenever informative to do so (typically when N per group is less than 10)
- For each experiment, you should identify the statistical tests used, exact values of N, definitions of center, methods of multiple test correction, and dispersion and precision measures (e.g., mean, median, SD, SEM, confidence intervals; and, for the major substantive results, a measure of effect size (e.g., Pearson's r, Cohen's d)
- Report exact p-values wherever possible alongside the summary statistics and 95% confidence intervals. These should be reported for all key questions and not only when the p-value is less than 0.05.

Please outline where this information can be found within the submission (e.g., page numbers or figure legends), or explain why this information doesn't apply to your submission:

eLife Sciences Publications, Ltd is a limited liability non-profit non-stock corporation incorporated in the State of Delaware, USA, with company number 5030732, and is registered in the UK with company number FC030576 and branch number BR015634 at the address 1st Floor, 24 Hills Road, Cambridge CB2 1JP | August 2014

eLIFE
1st Floor
24 Hills Road
Cambridge CB2 1JP, UK
P 01223 855340
W elifesciences.org
T @elife

(For large datasets, or papers with a very large number of statistical tests, you may upload a single table file with tests, Ns, etc., with reference to page numbers in the manuscript.)

Standard statistical analysis methods are explained and justified on pages 42-43. Conversion of raw reads and bisulfite conversions to normalized bisDRIP-seq scores is described and explained on pages 30-33. Calculation of bisDRIP-seq scores for regions is described on pages 34 and 35. Monte Carlo simulations were described on pages 35-36 and Figure 1 – figure supplement 1.

Raw data is included in Figure 1 – figure supplement 1 to illustrate a read that has bisulfite conversions.

The statistical tests used, exact values of N, definitions of center, methods of multiple test correction, and dispersion and precision measures are described in the figure legends of:

Figure 1 – figure supplement 1C: Wilcoxon signed-rank test, n = 29561 genomic regions, non-parametric test
Figure 1 – figure supplement 1E: Wilcoxon signed-rank test, n = 29561 genomic regions, non-parametric test
Figure 2A: Wilcoxon signed-rank test, n = 60017 regions, non-parametric test
Figure 2C: Wilcoxon signed-rank test, n = 4895 promoter regions, non-parametric test
Figure 2 – figure supplement 1A: Wilcoxon signed-rank test, n = 60017 regions, non-parametric test
Figure 2 – figure supplement 1B: Spearman's test using aymptotic t approximation, n = 78218 promoter regions, non-parametric test
Figure 2 – figure supplement 1C: Wilcoxon signed-rank test, n = 60017 promoter regions, non-parametric test
Figure 3A: Wilcoxon signed-rank test, n = 13 samples, non-parametric test. Multiple-hypothesis test correction was performed using a Bonferroni approach and initial p-value were multiplied by the total number of hypotheses (2001 nucleotide positions)
Figure 3 – figure supplement 2B: Wilcoxon signed-rank test, n = 3020 promoter regions, non-parametric test

We also describe statistical tests on:
page 7: Spearman's test using asymptotic t approximation, n = 78218 promoter regions, non-parametric test.
pages 8-9: Spearman's test using asymptotic t approximation, n = 78218 promoter regions, non-parametric test.

eLife Sciences Publications, Ltd is a limited liability non-profit non-stock corporation incorporated in the State of Delaware, USA, with company number 5030732, and is registered in the UK with company number FC030576 and branch number BR015634 at the address 1st Floor, 24 Hills Road, Cambridge CB2 1JP | August 2014

eLIFE
1st Floor
24 Hills Road
Cambridge CB2 1JP, UK
P 01223 855340
W elifesciences.org
T @elife

Additional data files ("source data")

- We encourage you to upload relevant additional data files, such as numerical data that are represented as a graph in a figure, or as a summary table
- Where provided, these should be in the most useful format, and they can be uploaded as "Source data" files linked to a main figure or table
- Include model definition files including the full list of parameters used
- Include code used for data analysis (e.g., R, MatLab)
- Avoid stating that data files are "available upon request"

Please indicate the figures or tables for which source data files have been provided:

(1) Figure 1B is based on Source_data_file_1.xls
(2) In Figure 4, the genomic location of exon-intron junctions are included in Source_data_file_2.txt
(3) Figure 5A and 5B and Table 1 are based on Source_data_file_3.txt

Normalized bisDRIP-seq scores for Figures 1-6 were produced from read sequence data using processingbisDRIPseqreads.py

Metaplot analysis for Figures 3-5 was performed using bisDRIPseqmetaplotanalysis.py

Region scores for Figures 2 and 5 were calculated using regionbisDRIPseqscores.py

Source code for Monte Carlo simulations are included in Monte_Carlo_for_shuffling_bisDRIPseq_scores.py and Monte_Carlo_random_assign_reads_to_regions.py

eLife Sciences Publications, Ltd is a limited liability non-profit non-stock corporation incorporated in the State of Delaware, USA, with company number 5030732, and is registered in the UK with company number FC030576 and branch number BR015634 at the address 1st Floor, 24 Hills Road, Cambridge CB2 1JP | August 2014
